# Individual and combined effects of the GSTM1, GSTT1, and GSTP1 polymorphisms on type 2 diabetes mellitus risk: A systematic review and meta-analysis

**DOI:** 10.3389/fgene.2022.959291

**Published:** 2022-11-07

**Authors:** Liang-shu Liu, Di Wang, Ru Tang, Qi Wang, Lu Zheng, Jian Wei, Yan Li, Xiao-feng He

**Affiliations:** ^1^ Changzhi Medical College, Changzhi, Shanxi, China; ^2^ Department of Endocrinology, Heping Hospital Affiliated to Changzhi Medical College, Changzhi, Shanxi, China; ^3^ Department of Epidemiology, School of Public Health to Southern Medical University, Guangzhou, Guangdong, China; ^4^ Institute of Evidence-Based Medicine, Heping Hospital Affiliated to Changzhi Medical College, Changzhi, China

**Keywords:** meta-analysis, genetic polymorphism, GSTM1, GSTP1, GSTT1, T2DM

## Abstract

**Backgrounds:** Compared with previously published meta-analyses, this is the first study to investigate the combined effects of glutathione-S-transferase polymorphisms (GSTM1, GSTT1 and GSTP1 IIe105Val) and type 2 diabetes mellitus (T2DM) risk; moreover, the credibility of statistically significant associations was assessed; furthermore, many new original studies were published.

**Objectives:** To determine the relationship between GSTM1, GSTT1, and GSTP1 polymorphisms with T2DM risk.

**Methods:** PubMed, Embase, Wanfang, and China National Knowledge Infrastructure Databases were searched. We quantify the relationship using crude odds ratios and their 95% confidence intervals Moreover, the Venice criteria, false-positive report probability (FPRP), and Bayesian false discovery probability (BFDP) were used to validate the significance of the results.

**Results:** Overall, significantly increased T2DM risk was found between individual and combined effects of GSTM1, GSTT1, and GSTP1 polymorphisms on T2DM risk, but, combined effects of the GSTT1 and GSTP1 polymorphisms was not statistically significant. GSTT1 gene polymorphism significantly increases the risk of T2DM complications, while GSTM1 and GSTP1 polymorphisms had no statistical significance. The GSTM1 null genotype was linked to a particularly increased risk of T2DM in Caucasians; the GSTT1 null genotype was connected to a significantly higher risk of T2DM in Asians and Indians; and the GSTP1 IIe105Val polymorphism was related to a substantially increased T2DM risk in Indians. Moreover, the GSTM1 and GSTT1 double null genotype was associated with substantially increased T2DM risk in Caucasians and Indians; the combined effects of GSTM1 and GSTP1 polymorphisms was associated with higher T2DM risk in Caucasians. However, all significant results were false when the Venice criteria, FPRP, and BFDP test were used (any FPRP >0.2 and BFDP value >0.8).

**Conclusion:** The current analysis strongly suggests that the individual and combined effects of GSTM1, GSTT1 and GSTP1 polymorphisms might not be connected with elevated T2DM risk.

## Introduction

Insulin resistance, excessive or improper glucagon secretion, and insufficient insulin secretion are symptoms of type 2 diabetes mellitus (T2DM), a chronic endocrine metabolic disorder. According to the newly released International Diabetes Federation Diabetes Atlas (10th edition), there are an estimated 537 million people living with diabetes in the world now, with that number expected to rise to 784 million by 2,045 based on current trends. In recent years, the incidence of diabetic complications, such as diabetic nephropathy and diabetic cardiovascular disease, has gradually increased. With the increase of people’s lifestyle such as poor diet, sedentary and inactive, the prevalence of T2DM will continue to rise, which will undoubtedly bring a huge burden on human health and medical expenditure (https://diabetesatlas.org/en/.). T2DM pathogenesis is complicated and diverse, involving both environmental and genetic risk factors. Currently, antioxidant and detoxification gene polymorphisms are believed to play a significant role in the risk of T2DM and related complications (Wu et al., 2014; Pahwa et al., 2017; Dendup et al., 2018).

Oxidative stress is the result of imbalance of reactive oxygen species generation and clearance, which is closely related to insulin resistance and functional impairment of islet β-cells. GST is a superfamily protease encoded by multiple genes with antioxidant and detoxification functions (Tangvarasittichai, 2015; Vats et al., 2015). It catalyzes the formation of thioether bonds between glutathione and electrophiles, thereby catalyzing the reduction of hydrogen peroxide, reducing fatty acids, phospholipids and DNA bases to corresponding alcohols, and scavenging lipid free radicals and hydrogen peroxide, thus reducing body damage (Hayes et al., 2005; Tabatabaei-Malazy et al., 2017). GSTM1 and GSTT1 gene locus has been mapped on chromosome 1p13.3 and 22q11.2, respectively, resulting in the reduced activity of a functional gene product (Hayes and Strange, 2000; Gonul et al., 2012). GSTP1 gene polymorphism is a single nucleotide polymorphism, a codon 105 A-G mutation at exon 5 in GSTP1 polymorphism leads to change in isoleucine (IIe) to valine (Val), is linked to a change in decreased enzymatic activity (Zimniak et al., 1994; Mastana et al., 2013). Thus, these three gene mutations may increase the risk of T2DM based on biological effects.

Independent and combined effects of GSTM1, GSTT1 and GSTP1 IIe105Val polymorphisms with the risk of T2DM have been described in a number of original investigations, and the association of GSTs gene polymorphisms with T2DM complications has also been reported. Of note, these results were contradictory. Five published meta-analyses (Tang et al., 2013; Yi et al., 2013; Zhang et al., 2013; Saadat, 2017; Nath et al., 2019) reported the GSTM1, GSTT1 and GSTP1 IIe105Val polymorphisms with T2DM risk. Three published meta-analyses have investigated the association between GSTs gene polymorphisms and T2DM complications (Orlewski and Orlewska, 2015; Sun et al., 2015; Nath et al., 2019). Yet, their studies had certain limitations. This could be due to the limited sample size or the lack of quality assessments of the literature. More importantly, past meta-analyses did not assess the reliability of positive results. As a result, we conducted a systematic review of the literature and a latest meta-analysis to assess whether GSTM1, GSTT1, and GSTP1 gene contribute to the risk of T2DM and related complications. We are also investigated the effect of gene-gene interactions on T2DM risk.

## Materials and methods

### Search strategy

The present study followed the Preferred Reporting Items for Systematic Review and Meta-Analysis (PRISMA) group’s guidelines. The PubMed, Embase, Wan-fang, and China National Knowledge Infrastructure (CNKI) databases were used to conduct the literature search (deadline to 16 May 2022). The following was utilized as a search strategy (polymorphism OR variant OR variation OR mutation OR SNP OR genome-wide association study OR genetic association study OR genotype OR allele) and (diabetes OR mellitus OR diabetes mellitus) AND (glutathione S-transferase M1 OR GSTM1 OR glutathione S-transferase T1 OR GSTT1 OR glutathione S-transferase P1 OR GSTP1). References of the retrieved articles and review articles on this topic were also carefully examined in additional suitable studies. If necessary, the respective authors were contacted by e-mail. Moreover, there were no restrictions or limitations on language.

### Inclusion and exclusion criteria

The inclusion criteria were: 1) case–control or cohort design; 2) studies must examine the correlation between the individual and combined effects of GST polymorphisms with T2DM or T2DM complication risk; 3) studies with odds ratio (OR) and their 95% confidence interval (CI), or enough data to calculate these numbers. Exclusion criteria were: 1) case reports, reviews, commentaries and meta-analyses; 2) studies that did not give genotype frequencies or could not determine the number of genotypes and alleles; 3) overlapping data or incomplete data.

### Data extraction

Two investigators extracted and double-checked data. When their conclusions differed, through discussion were reached a consensus. The first author’s name, year, country, source of control, match, race and other information were recorded, among which race was divided into “Asian,” “African,” “European,” “Caucasian,” “Indian” and “Mixed.”

### Quality score assessment

The quality assessment scale was independently assessed by two investigators (Supplementary Table S2). The total score was 18 points, studies scoring >12 were high, while scores of ≤12 were regarded as low quality in this meta-analysis. The scale was influenced by selection (two points), control and case source (four points), diagnostic criteria for T2DM (two points), Ascertainment of control (two points), matching (two points), Genotyping examination (two points), Association assessment (two points), and Hardy-Weinberg balance (HWE).

### Statistical analysis

Stata 12.0 software (Stata Corporation, College Station, TX) was used to calculate all statistical analyses. The strength of the association of GSTM1, GSTT1, and GSTP1 polymorphisms with T2DM and T2DM complications risk were estimated by calculating the crude OR and their 95% CI. We used the Chi-square-based Q-test and I2 value to evaluate heterogeneity (Higgins et al., 2003). The random-effects model was used when *p* was less than 0.10 and/or I2 was larger than 50%. if not, the fixed-effects model was applied (DerSimonian and Laird, 2015). Furthermore, a meta-regression analysis was used to investigate sources of heterogeneity (Baker et al., 2009). Subgroups were carried out based on ethnicity, control source, control type, and matching. Sensitivity analysis was performed using the following methods: 1) excluded one study at a time; 2) removed low-quality or Hardy–Weinberg Disequilibrium (HWD) studies; 3) retained only high quality and HWE research. HWE was evaluated by Chi-square goodness-of-fit test, *p* < 0.05 was defined as HWE, otherwise as HWD. To determine publication bias, Begg’s funnel plot (Begg and Mazumdar, 1994) and Egger’s test (Egger et al., 1997) were conducted. If publication bias existed, the missing studies would be filled employing a nonparametric “trim and fill” method (Dual and Tweedie, 2000).

### The credibility of genetic association

To assess the reliability of statistically significant correlations, the false-positive report probability (FPRP) (Wacholder et al., 2004), Bayesian False Discovery Probability (BFDP) (Wakefield, 2008), and the Venice standard (Ioannidis et al., 2008) were used. The FPRP and BFDP values were set to 0.2 and 0.8, respectively, and statistically significant correlations satisfying the following criteria were defined as “positive results” (Theodoratou et al., 2012): 1) at least two genetic models were statistically important (GSTM1 and GSTT1 polymorphisms are exempt from this requirement); 2) FPRP <0.2 and/or BFDP <0.8; 3) statistical power >80%; and 4) I2 < 50%. Otherwise, a positive result is considered less reliable.

## Results

### Search results and study characteristics

**FIGURE 1:**
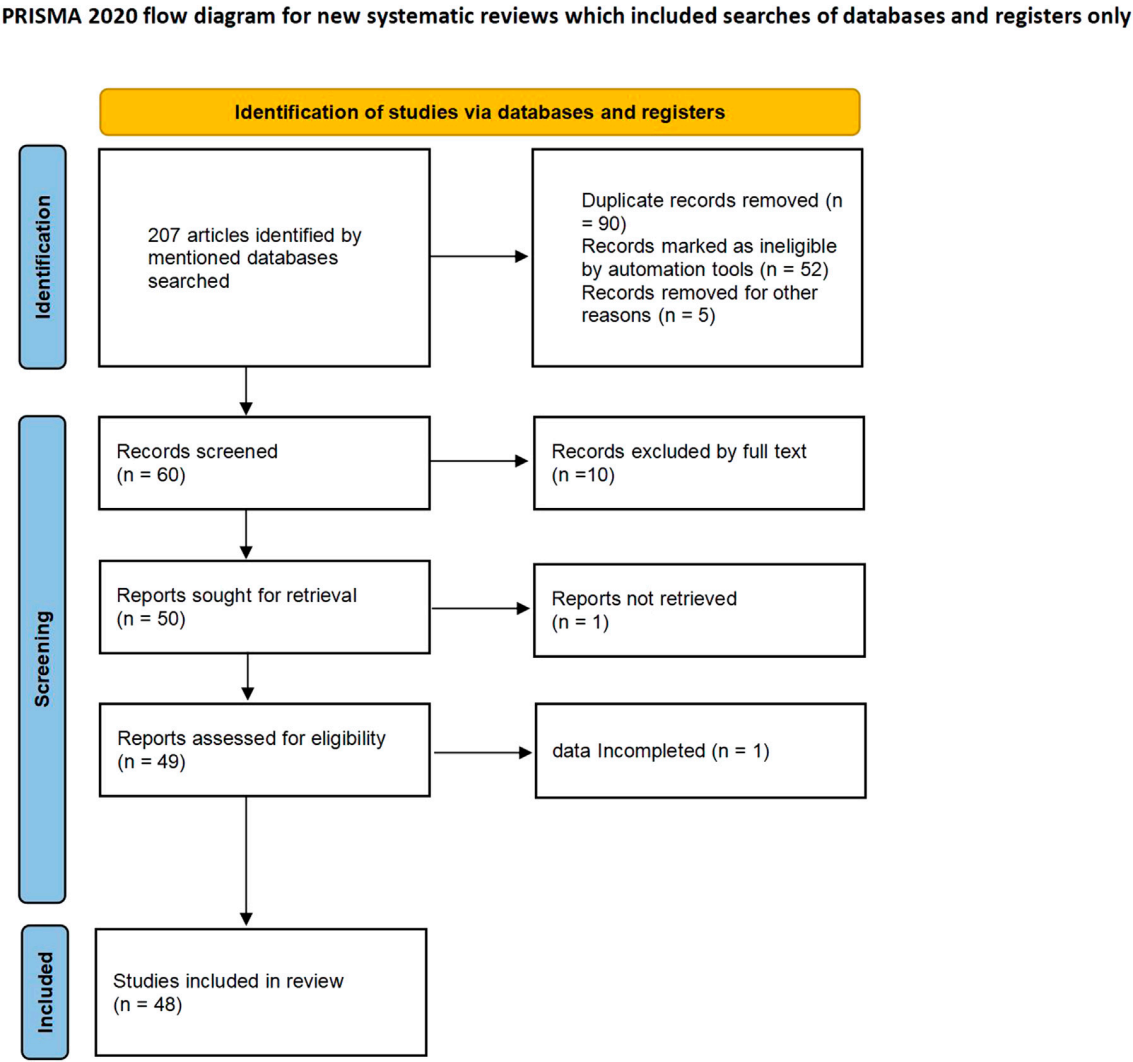
Flow diagram for searching studies in the current meta-analysis.

207 records were returned by PubMed, EMBASE, Wan-fang and CNKI databases. By carefully reading titles and abstracts, 147 publications were eliminated. Two authors independently read the remaining 60 publications in their entirety. 12 is excluded from the full text. As a result, 48 studies (Fujita et al., 2000; Yang et al., 2004; Hayek et al., 2006; Wang et al., 2006; Hori et al., 2007; Yalin et al., 2007; Oniki et al., 2008; Nowier et al., 2009; Tiwari et al., 2009; Bid et al., 2010; Datta et al., 2010; Amer et al., 2011; Ramprasath et al., 2011; Tsai et al., 2011; Amer et al., 2012; Cilenšek et al., 2012; Gonul et al., 2012; Jana and Petrovic., 2012; Moasser et al., 2012; Dadbinpour et al., 2013; Grubisa et al., 2013; Mastana et al., 2013; Pinheiro et al., 2013; Vats et al., 2013; Abbasi et al., 2014; Al-Badran and Al-Mayah, 2014; Moasser et al., 2014; Purkait et al., 2014; Rao et al., 2014; Raza et al., 2014; Afrand et al., 2015; Rasheed et al., 2015; Stoian et al., 2015; Zaki et al., 2015; Etemad et al., 2016; Mergani et al., 2016; Mir et al., 2016; Rasheed et al., 2016; Ahmed and Al-Bachary, 2017; Azarova et al., 2018; de Lima et al., 2018; Abbas et al., 2019; Osman et al., 2019; Klusek et al., 2020; Pourkeramati et al., 2020; Gusti et al., 2021; Albeladi et al., 2022; Jamil et al., 2022) were included (Figure 1). There were 28 articles (involving 4,878 cases and 4,621 controls, Table 1) on the GSTM1 present/null polymorphism, 28 articles (involving 4,710 cases and 4,471 controls, Table 2) on the GSTT1 present/null polymorphism, 24 studies on the GSTP1 IIe105Val polymorphism (including 4,297 cases and 4,244 controls, Table 3),17 studies including 3,035 cases and 3,241 controls concerned the combined GSTM1 and GSTT1 gene (Table 4), nine studies with 1,953 cases and 1,905 controls concerned the combined GSTM1 and GSTP1 gene (Table 5), nine papers for the combined of GSTT1 and GSTP1 gene studies, 1,953 cases and 1,905 controls were involved (Table 6), there were seven studies for the combined of GSTM1, GSTT1, and GSTP1 gene, included 1,299 cases and 1,334 controls (Table 7). Finally, there were five high-quality investigations on GSTM1, GSTT1 and GSTP1, the combined of GSTM1 and GSTT1 had four high-quality investigations, three high-quality researches combined of GSTM1 and GSTP1 were found, the combined of GSTT1 and GSTP1 had two high-quality studies, as well as two high-quality research on the combined effects of GSTM1, GSTT1, and GSTP1 (Supplementary Table S3). 17 studies on the correlation between GSTs gene and complications of T2DM (Supplementary Table S8).

**TABLE 1:** Meta-analysis of the association of GSTM1 polymorphism with risk of T2DM.

Variable	*n*	Cases/Controls	Test of association	Test of heterogeneity		Model
			Or (95%CI)	*P*_*h*_	*I* ^*2*^ (%)	
Overall	28	4,878/4,621	1.36 (1.08–1.72)	<0.001	83.8	Random-effect
Ethnicity						
Indian	8	1,689/1,407	1.37 (0.85–2.20)	<0.001	88.7	Random-effect
Asian	5	647/1,040	1.46 (0.81–2.64)	<0.001	86.3	Random-effect
Caucasian	13	2,194/1940	1.44 (1.04–2.01)	<0.001	79.3	Random-effect
Source of control						
HB	25	4,330/4,162	1.37 (1.08–1.74)	<0.001	83.1	Random-effect
PB	3	521/459	1.29 (0.45–3.73)	<0.001	91.1	Random-effect
Type of control						
HC	25	4,683/3,890	1.24 (0.99–1.56)	<0.001	80.9	Random-effect
NDC	3	275/731	1.97 (1.01–3.86)	0.009	78.9	Random-effect
Matching						
Yes	18	3,769/3,222	1.36 (1.02–1.82)	<0.001	87.3	Random-effect
No	10	1,109/1,399	1.36 (0.93–1.99)	<0.001	71.5	Random-effect
geographic region						
Asia	19	3,584/3,254	1.48 (1.13–1.99)	<0.001	74.0	Random-effect
Africa	3	183/167	1.10 (0.48–2.51)	0.022	68.8	Random-effect
Europe	4	1,011/966	1.29 (0.93–1.78)	<0.001	83.4	Random-effect
Sensitivity analysis						
Quality score>12						
Overall	5	1,633/1,399	1.23 (0.65–2.32)	<0.001	93.5	Random-effect
Ethnicity						
Indian	3	933/779	1.58 (0.60–4.19)	<0.001	94.9	Random-effect
Caucasian	2	700/620	0.84 (0.39–1.81)	0.012	84.3	Random-effect
geographic region						
Asia	3	933/779	1.58 (0.60–4.19)	<0.001	94.9	Random-effect
Source of control						
HB	3	1,212/990	1.21 (0.52–2.85)	<0.001	94.6	Random-effect
PB	2	421/409	1.22 (0.26–5.70)	<0.001	95.5	Random-effect
Matching and Quality score>12						
Overall	5	1,633/1,399	1.23 (0.65–2.32)	<0.001	93.5	Random-effect

**TABLE 2:** Meta-analysis of the association of GSTT1 polymorphism with risk of T2DM.

Variable	n	Cases/Controls	Test of association	Test of heterogeneity		Model
			OR (95% CI)	*P*_h_	*I* ^2^ (%)	
Overall	28	4710/4471	**1.45 (1.18–1.79)**	<0.001	71.5	Random-effect
Ethnicity						
Indian	7	1582/1355	**1.71 (1.20–2.43)**	<0.001	66.9	Random-effect
Asian	4	562/842	**1.46 (1.23–1.88)**	0.949	0.0	Random-effect
Caucasian	15	2345/1355	1.21 (0.86–1.70)	<0.001	75.8	Random-effect
Source of control						
HB	24	4138/3962	**1.60 (1.33–1.92)**	<0.001	57.4	Random-effect
PB	4	572/509	0.69 (0.23–2.06)	<0.001	91.1	Random-effect
Type of control						
HC	25	4420/3888	**1.43 (1.13–1.80)**	<0.001	74.3	Random-effect
NDC	3	290/583	**1.63 (1.15–2.30)**	0.620	0.0	Random-effect
Matching						
Yes	19	3793/3218	1.28 (0.98–1.67)	<0.001	77.5	Random-effect
No	9	917/1199	**1.98 (1.36–2.89)**	0.036	0.0	Random-effect
geographic region						
Asia	18	3363/3204	**1.55 (1.16-2.08)**	<0.001	84.8	Random-effect
Africa	4	283/217	0.63 (0.43-1.00)	0.622	0.0	Random-effect
Europe	4	1011/966	1.50 (0.91-2.46)	<0.001	83.4	Random-effect
Sensitivity analysis						
Quality score >12						
Overall	5	1633/1399	1.34 (0.79–2.28)	<0.001	87.0	Random-effect
Ethnicity						
Indian	3	933/779	1.69 (0.86–3.31)	0.001	86.1	Random-effect
Caucasian	2	700/620	0.92 (0.29–2.92)	0.001	90.6	Random-effect
geographic region						
Asia	3	933/779	1.69 (0.86-3.31)	0.001	86.1	Random-effect
Source of control						
HB	3	1212/990	1.66 (0.86–3.23)	0.001	86.8	Random-effect
PB	2	421/409	0.94 (0.28–3.09)	0.001	90.7	Random-effect
Matching and quality score >12						
Overall	5	1633/1399	1.34 (0.79–2.28)	<0.001	87.0	Random-effect

HB = hospital-based studies, PB = population-based studies, HC = Healthy control, NDC = Non-diabetic controls

**TABLE 3:** Meta-analysis of the association of GSTP1 polymorphism with risk of T2DM.

Variable	*n* (Cases/Controls)	Val/Val vs. IIe/IIe		IIe/Val vs. IIe/IIe		Val/Val vs. IIe/IIe + IIe/Val		Val/Val + IIe/Val vs. IIe/IIe		Val vs. IIe	
		Or (95%CI)	*P*_*h*_ */I* ^*2*^ (%)	Or (95%CI)	*P*_*h*_ */I* ^*2*^ (%)	Or (95%CI)	*P*_*h*_ */I* ^*2*^ (%)	Or (95%CI)	*P*_*h*_ */I* ^*2*^ (%)	Or (95%CI)	*P*_*h*_ */I* ^*2*^ (%)
Overall	24 (4,297/4,244)	1.37 (0.99–1.88)	0.002/53.2	1.24 (1.02–1.50)	0.000/70.0	1.27 (0.98–1.61)	0.104/29.2	1.27 (1.05–1.53)	0.000/72.1	1.22 (1.05–1.42)	0.000/71.1
Ethnicity											
Caucasian	14 (2049/2026)	1.11 (0.76–1.62)	0.057/42.7	1.13 (0.88–1.46)	0.000/67.6	1.00 (0.79–1.26)	0.534/0.0	1.16 (0.90–1.48)	0.000/70.1	1.11 (0.92–1.33)	0.000/64.1
Indian	6 (1751/1,328)	2.17 (1.59–2.96)	0.441/0.0	1.54 (1.22–1.94)	0.081/48.9	1.84 (1.37–2.47)	0.431/0.0	1.61 (1.32–1.97)	0.167/36.0	1.47 (1.27–1.70)	0.238/26.3
Source of control											
HB	18 (3,605/3,182)	1.24 (0.89–1.73)	0.007/52.9	1.16 (0.91–1.47)	0.000/75.3	1.14 (0.90–1.45)	0.224/19.7	1.18 (0.93–1.49)	0.000/77.6	1.33 (0.96–1.34)	0.000/74.0
PB	3 (496/902)	2.56 (1.47–4.44)	–	1.42 (0.98–2.07)	0.220/34.1	2.29 (1.36–3.85)	–	1.45 (1.13–1.85)	0.540/0.0	1.48 (1.20–1.83)	0.566/0.0
Type of control											
HC	20 (3,652/3,394)	1.38 (0.96–1.98)	0.001/59.3	1.34 (1.09–1.64)	0.000/69.5	1.23 (0.94–1.63)	0.046/39.8	1.37 (1.11–1.69)	0.000/73.5	1.28 (1.09–1.51)	0.000/72.5
NDC	4 (645/850)	1.28 (0.65–2.51)	0.635/0.0	0.77 (0.52–1.15)	0.168/44.0	1.41 (0.73–2.72)	0.790/0.0	0.89 (0.64–1.24)	0.171/40.1	0.91 (0.66–1.25)	0.174/42.9
Matching											
Yes	14 (3,080/2,847)	1.49 (1.01–2.20)	0.001/63.2	1.35 (1.00–1.72)	0.000/74.7	1.32 (0.99–1.77)	0.051/42.7	1.40 (1.09–1.79)	0.000/77.8	1.31 (1.09–1.57)	0.000/76.3
No	10 (1,217/1,397)	1.09 (0.61–1.96)	0.228/25.2	1.04 (0.76–1.43)	0.021/55.5	1.12 (0.72–1.76)	0.420/1.3	1.07 (0.81–1.42)	0.020/54.4	1.07 (0.83–1.36)	0.025/54.3
Geographic region											
Asia	15 (3,967/3,513)	1.76 (1.24–2.50)	0.112/32.9	1.38 (1.10–1.73)	0.000/64.9	1.52 (1.45–2.00)	0.299/14.1	1.42 (1.34–1.78)	0.000/66.5	1.32 (1.12–1.56)	0.001/60.4
Europe	5 (1,151/1,066)	0.99 (0.62–1.54)	0.124/44.6	0.88 (0.68–1.14)	0.140/42.2	1.00 (0.71–1.43)	0.269/22.8	0.92 (0.68–1.24)	0.036/61.1	0.98 (0.77–1.26)	0.016/67.1
HWE											
Yes	4 (609/556)	1.34 (0.51–3.51)	0.038/64.5	1.52 (0.81–2.84)	0.003/78.4	1.27 (0.62–2.61)	0.162/41.6	1.50 (0.82–2.75)	0.003/79.0	1.38 (0.88–2.17)	0.005/77.0
No	19 (3,625/3,193)	1.35 (0.96–1.90)	0.007/51.7	1.19 (0.97–1.43)	0.000/66.9	1.24 (0.95–1.60)	0.121/30.3	1.23 (1.00–1.51)	0.000/71.3	1.19 (1.01–1.40)	0.000/69.6
Sensitivity analysis											
HWD											
Overall	19 (3,625/3,193)	1.35 (0.96–1.90)	0.007/51.7	1.19 (0.97–1.43)	0.000/66.9	1.24 (0.95–1.60)	0.121/30.3	1.23 (1.00–1.51)	0.000/71.3	1.19 (1.01–1.40)	0.000/69.6
Ethnicity											
Indian	4 (1,303/899)	2.17 (1.15–3.12)	0.639/0.0	1.41 (1.06–1.86)	0.111/50.1	1.88 (1.33–2.65)	0.560/0.0	1.52 (1.16–1.99)	0.116/49.3	1.44 (1.17–1.78)	0.117/49.0
Caucasian	13 (1989/1986)	1.06 (0.72–1.55)	0.065/42.6	1.14 (0.88–1.47)	0.000/65.9	0.97 (0.76–1.23)	0.456/0.0	1.15 (0.89–1.49)	0.000/68.9	1.10 (0.92–1.32)	0.002/62.2
geographic region											
Asia	11 (2060/1778)	1.63 (1.07–2.50)	0.091/40.0	1.22 (0.94–1.59)	0.001/66.3	1.44 (1.03–2.01)	0.253/20.7	1.27 (0.97–1.66)	0.000/69.3	1.21 (0.99–1.46)	0.003/62.9
Europe	5 (1,151/1,066)	0.99 (0.62–1.54)	0.124/44.6	0.88 (0.68–1.14)	0.140/42.2	1.00 (0.71–1.43)	0.269/22.8	0.92 (0.68–1.24)	0.036/61.1	0.98 (0.77–1.26)	0.016/67.1
Source of control											
HB	13 (2,971/2,468)	1.20 (0.84–1.71)	0.021/49.7	1.17 (0.91–1.51)	0.000/72.3	1.10 (0.86–1.40)	0.311/13.3	1.20 (0.93–1.54)	0.000/75.9	1.14 (0.95–1.37)	0.000/72.4
PB	2 (433/497)	2.56 (1.47–4.44)	–	1.42 (0.98–2.07)	0.218/34.1	2.29 (1.36–3.85)	–	1.51 (1.15–1.99)	0.412/0.0	1.48 (1.20–1.83)	0.566/0.0
Type of control											
HC	17 (3,963/3,999)	1.43 (0.90–2.20)	0.001/62.6	1.32 (1.05–1.67)	0.000/68.6	1.21 (0.89–1.65)	0.053/42.4	1.37 (1.07–1.75)	0.000/73.8	1.28 (1.06–1.55)	0.000/73.4
NDC	3 (582/445)	1.28 (0.65–2.51)	0.635/0.0	0.77 (0.52–1.15)	0.168/44.0	1.41 (0.73–2.72)	0.790/0.0	0.81 (0.54–1.22)	0.142/48.9	0.91 (0.66–1.25)	0.174/42.9
Matching											
Yes	12 (2,632/2,418)	1.40 (0.92–2.14)	0.002/65.5	1.24 (0.94–1.64)	0.000/75.6	1.26 (0.93–1.72)	0.067/43.7	1.32 (1.01–1.74)	0.000/78.3	1.26 (1.03–1.55)	0.000/77.2
No	7 (1,165/1826)	1.14 (0.61–2.12)	0.321/14.5	1.03 (0.77–1.38)	0.115/41.5	1.15 (0.65–2.06)	0.350/10.3	1.05 (0.79–1.39)	0.112/41.8	1.04 (0.83–1.31)	0.154/36.0
Quality score>12											
Overall	5 (1,645/1,487)	1.64 (0.76–3.57)	0.001/81.9	1.39 (0.99–1.96)	0.002/76.8	1.46 (0.77–2.76)	0.006/75.8	1.47 (1.00–2.15)	0.000/82.5	1.39 (1.01–1.93)	0.000/85.0
Ethnicity											
Caucasian	2 (712/708)	0.72 (0.48–1.10)	–	1.21 (0.57–2.55)	0.012/84.2	0.77 (0.52–1.16)	–	1.19 (0.55–2.59)	0.008/85.7	1.15 (0.59–2.25)	0.011/84.5
Indian	3 (933/779)	2.21 (1.51–3.24)	0.444/0.0	1.53 (1.09–2.15)	0.103/56.1	1.88 (1.30–2.73)	0.357/2.80	1.65 (1.21–2.26)	0.123/52.3	1.54 (1.18–2.00)	0.087/59.0
Geographic region											
Asia	3 (933/779)	2.21 (1.51–3.24)	0.444/0.0	1.53 (1.09–2.15)	0.103/56.1	1.88 (1.30–2.73)	0.357/2.80	1.65 (1.21–2.26)	0.123/52.3	1.54 (1.18–2.00)	0.087/59.0
Source of control											
HB	3 (1,212/990)	1.35 (0.54–3.39)	0.010/78.4	1.38 (0.80–2.39)	0.001/86.2	1.16 (0.59–2.27))	0.060/64.4	1.43 (0.78–2.62)	0.000/89.5	1.33 (0.82–2.18)	0.000/90.1
PB	2 (443/497)	2.56 (1.47–4.44)	–	1.42 (0.98–2.07)	0.218/34.1	2.29 (1.36–3.85)	–	1.51 (1.15–1.99)	0.412/0.0	1.48 (1.20–1.83)	0.566/0.0

HB, hospital-based studies; PB, population-based studies; HC, healthy control; NDC, Non-diabetic controls.

**TABLE 4:** Meta-analysis of the combined effects of *GSTM1* present/null and *GSTT1* present/null on T2DM risk.

Variable	N (case/Control)	Model 1		Model 2		Model 3		Model 4		Model 5		Model 6	
		Or (95%CI)	*P*_h_/*I* ^2^ (%)	Or (95%CI)	*P*_h_/*I* ^2^ (%)	Or (95%CI)	*P*_h_/*I* ^2^ (%)	Or (95%CI)	*P*_h_/*I* ^2^ (%)	Or (95%CI)	*P*_h_/*I* ^2^ (%)	Or (95%CI)	*P*_h_/*I* ^2^ (%)
Overall	17 (3,035/3,241)	1.25 (0.82–1.92)	0.016/57.6	**2.28 (1.72–3.04)**	0.004/54.7	**2.19 (1.64–2.93)**	0.005/54.0	**1.41 (1.15–1.72)**	0.004/56.2	**1.89 (1.06–1.33)**	0.536/0.0	**1.75 (1.41–2.18)**	0.124/30.6
Ethnicity													
Caucasian	8 (1,638/1,364)	0.88 (0.50–1.55)	0.184/38.0	1.55 (0.99–2.44)	0.034/65.4	**2.12 (1.31–3.43)**	0.005/65.7	**1.47 (1.02–2.13)**	0.037/57.8	**1.25 (1.02–1.53)**	0.399/2.70	1.39 (0.98–1.99)	0.203/30.9
Indian	5 (969/988)	1.30 (0.60–2.83)	0.158/49.8	1.06 (0.44–2.54)	0.081/67.1	**2.39 (1.40–4.09)**	0.034/61.6	1.37 (0.93–2.01)	0.007/71.7	1.14 (0.97–1.35)	0.377/5.20	**2.08 (1.40–3.08)**	0.175/37.0
Source of control													
HB	14 (2,614/2,782)	1.18 (0.75–1.86)	0.014/60.3	1.28 (0.91–1.79)	0.007/64.0	**1.90 (1.41–2.56)**	0.033/46.6	**1.27 (1.03–1.58)**	0.220/50.6	**1.13 (1.00–1.29)**	0.570/0.0	**1.59 (1.29–1.96)**	0.339/10.8
PB	3 (521/459)	–	–	–	–	**3.92 (2.56–6.00)**	0.592/0.0	**2.04 (1.54–2.70)**	0.652/0.0	**1.40 (1.11–1.77)**	0.612/0.0	**2.68 (1.68–4.29)**	0.296/17.9
Type of control													
HC	15 (2,938/2,681)	1.16 (0.74–1.82)	0.019/58.4	1.25 (0.90–1.74)	0.010/62.2	**2.15 (1.58–2.92)**	0.004/56.4	**1.36 (1.10–1.69)**	0.008/54.1	**1.19 (1.05–1.33)**	0.460/0.0	**1.69 (1.37–2.08)**	0.204/23.1
Matching													
Yes	14 (2,847/2,615)	1.22 (0.74–2.01)	0.014/62.2	1.22 (0.83–1.79)	0.007/66.4	**2.34 (1.73–3.17)**	0.028/47.8	**1.40 (1.11–1.76)**	0.002/63.2	**1.18 (1.03–1.33)**	0.413/3.3	**1.90 (1.53–2.36)**	0.337/11.0
No	3 (288/626)	1.37 (0.45–4.17)	0.087/65.9	**1.65 (1.07–2.53)**	0.452/0.0	1.60 (0.63–4.05)	0.018/75.1	1.45 (0.98–2.14)	0.363/1.2	1.25 (0.89–1.77)	0.495/0.0	1.28 (0.70–2.35)	0.068/62.8
Geographic region													
Asia	11 (2,761/2,943)	1.18 (0.87–1.60)	0.368/7.9	**2.25 (1.70–3.0)**	0.136/34.0	1.42 (0.96–2.10)	0.006/66.9	**1.42 (1.10–1.81)**	0.007/60.4	**1.18 (1.04–1.34)**	0.457/0.0	**1.89 (1.53–2.27)**	0.552/0.0
Europe	3 (811/745)	–	–	2.52 (0.85–7.47)	0.013/77.0	–	–	–	–	–	–	–	–
Sensitivity analysis													
Quality score													
>12	4 (1,121/1,129)	–	–	–	–	**3.40 (2.42–4.79)**	0.669/0.0	**2.00 (1.54–2.59)**	0.616/0.0	**1.39 (1.12–1.74)**	0.61/0.0	**2.21 (1.40–3.50)**	0.299/17.1
Sample size													
>200	11 (2,451/2,644)	1.40 (0.87–2.25)	0.030/59.5	1.17 (0.86–1.59)	0.096/46.5	**2.30 (1.78–2.97)**	0.162/29.9	**1.34 (1.05–1.70)**	0.016/57.6	**1.16 (1.02–1.32)**	0.518/0.0	**1.80 (1.47–2.19)**	0.768/0.0
HWE													
Yes	15 (2,589/2,812)	1.31 (0.80–2.14)	0.014/60.3	**2.34 (1.68–3.26)**	0.006/55.6	**1.42 (1.04–1.94)**	0.040/52.3	**1.56 (1.25–1.94)**	0.040/46.1	**1.27 (1.11-1-46)**	0.667/0.0	**1.69 (1.31–2.19)**	0.118/34.0

Model 1 = M1 present/T1 null vs. M1 present/T1 present, Model 2 = M1 null/T1 present vs. M1 present/T1 present, Model 3 = M1 null/T1 null vs. M1 present/T1 present, Model 4 = All one risk genotypes vs. M1 present/T1 present, Model 5 = All risk genotypes vs. M1 present/T1 present, Model 6 = M1 null/T1 null vs. M1 present/T1 present + M1 present/T1 null + M1 null/T1 present, HB, hospital-based studies; PB, population-based studies.

The bold values indicated that these results are statistically significant.

**TABLE 5:** Meta-analysis of the combined effects of GSTM1 present/null and GSTP1 IIe105Val on T2DM risk.

Variable	Sample size	Model 1		Model 2		Model 3		Model 4		Model 5		Model 6	
		Or (95%CI)	*P*_*h*_ */I* ^*2*^ (%)	Or (95%CI)	*P*_*h*_ */I* ^*2*^ (%)	Or (95%CI)	*P*_*h*_ */I* ^*2*^ (%)	Or (95%CI)	*P*_*h*_ */I* ^*2*^ (%)	Or (95%CI)	*P*_*h*_ */I* ^*2*^ (%)	Or (95%CI)	*P*_*h*_ */I* ^*2*^ (%)
Overall	9 (1953/1905)	1.40 (0.94–2.08)	0.002/69.8	0.98 (0.58–1.65)	0.001/73.3	1.15 (0.82–1.50)	0.114/41.5	1.84 (1.13–3.00)	0.001/70.1	1.26 (0.89–1.76)	0.007/66.2	1.37 (1.03–1.82)	0.290/18.3
Ethnicity													
Caucasian	4 (836/767)	1.11 (0.60–2.07)	0.192/39.5	1.05 (0.49–2.22)	0.139/49.3	1.02 (0.67–1.56)	0.992/0.0	2.72 (1.78–4.16)	0.970/0.0	1.31 (0.87–1.96)	0.944/0.0	2.11 (1.29–3.44)	0.944/0.0
Indian	4 (869/938)	1.45 (0.74–2.87)	0.001/82.3	0.93 (0.44–1.96)	0.000/83.7	1.18 (0.78–1.77)	0.023/68.4	1.28 (0.53–3.06)	0.00/85.2	1.21 (0.71–2.04)	0.001/82.9	1.17 (0.86–1.59)	0.348/9.1
Source of control													
HB	8 (1,632/1,596)	1.23 (0.85–1.78)	0.025/58.5	0.91 (0.47–1.76)	0.001/75.7.3	1.05 (0.82–1.33)	0.378/6.1	1.67 (0.96–2.91)	0.005/67.5	1.11 (0.82–1.50)	0.133/40.8	1.36 (0.91–2.03)	0.198/31.7
Matching													
Yes	8 (1899/1854)	1.48 (0.97–2.25)	0.002/71.9	0.91 (0.52–1.61)	0.001/77.0	1.14 (0.84–1.55)	0.070/50.9	1.75 (1.04–2.97)	0.001/73.9	1.25 (0.85–1.82)	0.003/71.8	1.33 (0.98–1.79)	0.260/23.2
Sensitivity analysis													
Quality score													
>12	3 (1,021/1,029)	2.57 (1.53–4.31)	0.204/37.9	0.70 (0.16–2.98)	0.002/89.8	1.41 (0.88–2.56)	0.139/54.3	1.88 (0.84–4.18)	0.005/80.9	1.48 (0.69–3.19)	0.011/84.6	1.10 (0.58–2.06)	0.115/59.6
HWE													
Yes	7 (1,505/1,476)	1.72 (1.15–2.58)	0.037/57.8	0.90 (0.48–1.70)	0.008/70.8	1.30 (1.01–1.67)	0.400/1.20	2.24 (1.41–3.54)	0.050/54.8	1.42 (1.01–2.01)	0.112/46.6	1.48 (1.03–2.15)	0.217/30.7

Model 1 = M1 null/P1 IIe/IIe, vs. M1 present/P1 IIe/IIe, Model 2 = M1 present/P1 Val* vs. M1 present/P1 IIe/IIe, Model 3 = (M1 null/P1 IIe/IIe + M1 present/P1 Val*) vs. M1 present/P1 IIe/IIe; Model 4 = M1 null/P1 Val* vs. M1 present/P1 IIe/IIe, Model 5 = All risk genotypes vs. M1 present/P1 IIe/IIe, Model 6 = M1 null/P1 Val* vs. (M1 present/P1 IIe/IIe + M1 null/P1 IIe/IIe + M1 Present/P1 Val*), HB, hospital-based studies; PB, population-based studies.

**TABLE 6:** Meta-analysis of the combined effects of *GSTT1* present/null and *GSTP1* IIe105Val on T2DM risk.

Variable	Sample size	Model 1		Model 2		Model 3		Model 4		Model 5		Model 6	
		Or (95%CI)	*P*_h_/*I* ^2^	Or (95%CI)	*P*_h_/*I* ^2^	Or (95%CI)	*P*_h_/*I* ^2^	Or (95%CI)	*P*_h_/*I* ^2^	Or (95%CI)	*P*_h_/*I* ^2^	Or (95%CI)	*P*_h_/*I* ^2^
Overall	9 (1953/1905)	1.20 (0.72–1.20)	0.002/69.2	1.02 (0.68–1.53)	0.009/65.0	0.98 (0.73–1.31)	0.054/51.6	1.68 (0.90–3.15)	0.002/71.2	1.12 (0.79–1.59)	0.003/70.1	1.47 (0.94–2.28)	0.098/43.9
Ethnicity													
Caucasian	3 (236/247)	0.79 (0.34–1.86)	0.118/53.2	1.41 (0.92–2.17)	0.534/0.00	1.14 (0.78–1.66)	0.832/0.00	1.22 (0.48–3.10)	0.129/51.2	1.15 (0.80–1.66)	0.911/0.0	1.04 (0.45–2.42)	0.172/43.1
Indian	4 (869/938)	1.45 (0.59–3.55)	0.002/76.9	0.82 (0.46–1.48)	0.005/76.9	0.89 (0.56–1.42)	0.009/74.0	2.04 (0.92–4.52)	0.006/76.2	1.09 (0.63–1.90)	0.000/84.9	**1.75 (1.10–2.78)**	0.203/34.9
Source of control													
HB	7 (1,032/1,076)	0.85 (0.61–1.18)	0.434/0.00	1.01 (0.60–1.72)	0.005/70.5	0.90 (0.65–1.25)	0.099/46.0	1.46 (0.68–3.12)	0.004/70.6	1.03 (0.70–1.52)	0.010/67.0	1.36 (0.77–2.41)	0.083/48.7
Matching													
Yes	7 (1,299/1,334)	1.29 (0.67–2.49)	0.002/72.9	0.97 (0.63–1.50)	0.006/69.2	0.98 (0.71–1.36)	0.031/59.4	1.58 (0.78–3.20)	0.001/75.9	1.11 (0.76–1.64)	0.001/75.1	1.40 (0.85–2.30)	0.065/52.0
Sensitivity analysis													
Quality score													
≤12	5 (680/676)	0.85 (0.56–1.28)	0.303/17.5	1.30 (0.95–1.78)	0.598/0.00	1.07 (0.82–1.38)	0.1962/0.0	1.87 (0.92–3.81)	0.029/62.9	1.27 (1.00–1.61)	0.947/0.00	1.47 (0.77–2.81)	0.060/55.8
HWE													
Yes	6 (905/956)	1.30 (0.65–2.60)	0.001/74.6	1.00 (0.58–1.74)	0.004/74.3	0.96 (0.62–1.48)	0.015/67.6	1.24 (0.50–3.03)	0.002/76.2	1.02 (0.61–1.71)	0.001/78.9	1.23 (0.71–2.13)	0.147/41.2

Model 1 = *T1* null/*P1* IIe/IIe, vs. *T1* present/*P1* IIe/IIe, Model 2 = T1 present/P1 Val* vs. *T1* present/*P1* IIe/IIe, Model 3 = (*T1* null/*P1* IIe/IIe + *T1 p*resent/*P1* Val*) vs. *T1* present/*P1* IIe/IIe, Model 4 = *T1 n*ull*/P1 *Val* vs. *T1* present/*P1* IIe/IIe, Model 5 = All risk genotypes vs. *T1* present/*P1* IIe/IIe, Model 6 = T*1* null*/P1 *Val* vs. (T*1* present/*P1* IIe/IIe + T*1* null/*P1* IIe/IIe + T*1* Present/*P1* Val*), HB, hospital-based studies; PB, population-based studies.

The bold values indicated that these results are statistically significant.

**TABLE 7:** Meta-analysis of the combined effects of *GSTM1* present/null, *GSTT1* present/null and *GSTP1* present/null on T2DM risk.

Variable	Sample size	Model 1		Model 2		Model 3		Model 4		Model 5		Model 6		Model 7		Model 8		Model 9		Model 10	
		Or (95%CI)	*P*_h_/*I* ^2^ (%)	Or (95%CI)	*P*_h_/*I* ^2^ (%)	Or (95%CI)	*P*_h_/*I* ^2^ (%)	Or (95%CI)	*P*_h_/*I* ^2^ (%)	Or (95%CI)	*P*_h_/*I* ^2^ (%)	Or (95%CI)	*P*_h_/*I* ^2^ (%)	Or (95%CI)	*P*_h_/*I* ^2^ (%)	Or (95%CI)	*P*_h_/*I* ^2^ (%)	Or (95%CI)	*P*_h_/*I* ^2^ (%)	Or (95%CI)	*P*_h_/*I* ^2^ (%)
Overall	7 (1,299/1,334)	**1.47 (0.10–1.96)**	0.218/27.6	0.80 (0.55–1.14)	0.242/24.5	0.73 (0.44–1.23)	0.009/67.2	0.91 (0.70–1.19)	0.143/39.4	1.61 (0.82–3.17)	0.010/64.5	1.17 (0.75–1.82)	0.098/46.1	1.06 (0.42–2.66)	0.002/74.0	1.18 (0.83–1.08)	0.065/51.9	2.71 (1.56–4.72)	0.276/20.9	**2.69 (1.74–4.17)**	0.382/5.4
Sensitivity analysis																					
Quality score																					
>12	2 (421/509)	**1.81 (1.02–3.22)**	0.185/43.2	0.64 (0.17–2.40)	0.063/71.1	0.44 (0.30–0.64)	0.474/0.0	0.84 (0.38–1.86)	0.015/83.3	1.08 (0.45–2.60)	0.555/0.0	0.81 (0.54–1.22)	0.993/0.0	0.66 (0.37–1.18)	0.682/0.0	0.80 (0.56–1.120	0.870/0.0	**1.75 (1.00–3.07)**	0.697/0.0	**2.24 (1.32–3.80)**	0.502/0.0
HWE																					
Yes	5 (851/905)	**1.61 (1.15–2.25)**	0.256/24.8	0.76 (0.42–1.39)	0.140/42.2	0.59 (0.33–1.04)	0.096/52.7	0.89 (0.57–1.38)	0.071/57.3	2.02 (0.65–6.31)	0.004/74.2	1.32 (0.66–2.64)	0.028/67.2	0.59 (0.35–1.00)	0.796/0.0	1.01 (0.64–1.60)	0.128/47.2	**1.87 (1.12–3.11)**	0.757/0.0	**2.19 (1.35–3.54)**	0.577/0.0

Model 1 = M1 null/T1 present/P1 IIe/IIe, vs. M1 present/T1 present/P1 IIe/IIe, Model 2 = M1 present/T1 null/P1 IIe/IIe, vs. M1 present/T1 present/P1 IIe/IIe, Model 3 = M1 present/T1 present/P1 Val 1 vs. M1 present/T1 present/P1 IIe/IIe, Model 4 = all one high-risk genotype vs. vs. M1 present/T1 present/P1 IIe/IIe, Model 5 = M1 null/T1 null/P1 IIe/IIe, vs. M1 present/T1 present/P1 IIe/IIe, Model 6 = M1 null/T1 present/P1 Val 1 vs. M1 present/T1 present/P1 IIe/IIe, Model 7 = M1 present/T1 null/P1 Val1 vs. M1 present/T1 present/P1 IIe/IIe, Model 8 = all two high-risk genotype vs. M1 present/T1 present/P1 IIe/IIe, Model 9 = M1 null/T1 null/P1 Val 1 vs. M1 present/T1 present/P1 IIe/IIe, Model 10 = M1 null/T1 null/P1 Val 1 vs. M1 present/T1 present/P1 IIe/IIe + all one high-risk genotype + all two high-risk genotypes.

The bold values indicated that these results are statistically significant.

### Quantitative synthesis

Overall, the individuals who carried the GSTM1 null genotype had a significantly increased T2DM risk (OR = 1.36, 95% CI: 1.04–1.72, Table 1). When subgroup analyses were performed, the same substantial association were found in Caucasians (OR = 1.44, 95% CI: 1.04–2.01), hospital-based studies (OR = 1.37, 95% CI: 1.08–1.74), matching (OR = 1.36, 95% CI: 1.02–1.82), non-diabetic controls (OR = 1.97, 95% CI: 1.01–3.86), and Asia (OR = 1.48, 95% CI: 1.13–1.99).

Overall, a notable connection was discovered between the GSTT1 polymorphism and an increased risk of T2DM (OR = 1.45, 95% CI: 1.18–1.79, Table 2). Similarly, high correlation was found in subgroup analyses in Indians (OR = 1.71, 95% CI: 1.20–2.43), Asians (OR = 1.46, 95% CI: 1.23–1.88), hospital-based studies (OR = 1.60, 95% CI: 1.33–1.92), healthy controls (OR = 1.43, 95% CI: 1.13–1.80), non-diabetic controls (OR = 1.63, 95% CI: 1.15–2.30), non-matching (OR = 1.98, 95% CI: 1.36–2.89), and Asia (OR = 1.55, 95% CI: 1.16–2.08).

The GSTP1 polymorphism were also found to be associated with increased risk of T2DM (IIe/Val vs. IIe/IIe: OR = 1.24, 95% CI = 1.02–1.50, Val/Val + IIe/Val vs. IIe/IIe: OR = 1.27, 95% CI = 1.05–1.53 and Val vs. IIe: OR = 1.22, 95% CI = 1.05–1.42, Table 3). As for the subgroup analysis of populations in different Ethnicity, high associations were observed in Caucasians (Val/Val vs. IIe/IIe: OR = 2.17, 95% CI = 1.59–2.96, IIe/Val vs. IIe/IIe: OR = 1.54, 95% CI = 1.22–1.94, Val/Val vs. IIe/IIe + IIe/Val: OR = 1.84, 95% CI = 1.37–2.47, Val/Val + IIe/Val vs. IIe/IIe: OR = 1.61, 95% CI = 1.32–1.97, Val vs. IIe: OR = 1.47, 95% CI = 1.27–1.70). In addition, similar result was found in the population-based studies, healthy controls and Asia.

This study revealed a strong association combination of GSTM1 and GSTT1 polymorphisms and T2DM susceptibility in overall analysis (model 2: OR = 2.28, 95% CI = 1.72–3.04; model 3: OR = 2.19, 95% CI = 1.64–2.93; model 4: OR = 1.41, 95% CI = 1.15–1.72; model 5: OR = 1.89, 95% CI = 1.06–1.33; model 6: OR = 1.75, 95% CI = 1.41–2.18, Table 4). Furthermore, substantially association were showed in Caucasians (model 3: OR = 2.12, 95% CI = 1.31–3.43; model 4: OR = 1.47, 95% CI = 1.02–2.13; model 5: OR = 1.25, 95% CI = 1.02–1.53), Indians, hospital-based studies, population-based studies, healthy controls, non-matching, matching and Asia.

An increased risk of T2DM was yielded on the combined effects of both GSTM1 and GSTP1 (model 4: OR = 1.84, 95% CI = 1.13–3.00, model 6: OR = 1.37, 95% CI = 1.03–1.82, Table 5) in overall analysis. subgroups also showed an increased T2DM risk in Caucasians (model 4: OR = 2.72, 95% CI = 1.78–4.16, model 6: OR = 2.11, 95% CI = 1.29–3.44) and matching controls (model 4: OR = 1.75, 95% CI = 1.04–2.97).

Combinations of GSTT1 and GSTP1 polymorphism were not associated with T2DM risk in overall analysis. However, when subgroup analysis for ethnicity was stratified, important association were found in Indians (model 6: OR = 1.75, 95% CI = 1.10–2.78, Table 6).

In overall populations, combinations of GSTM1, GSTT1 and GSTP1 polymorphisms was considerably associated with T2DM risk (model 1: OR = 1.47, 95% CI = 1.10–1.96, model 9: OR = 2.71, 95% CI = 1.56–4.72, model 10: OR = 2.69, 95% CI = 1.74–4.17, Table 7).

**FIGURE 2:**
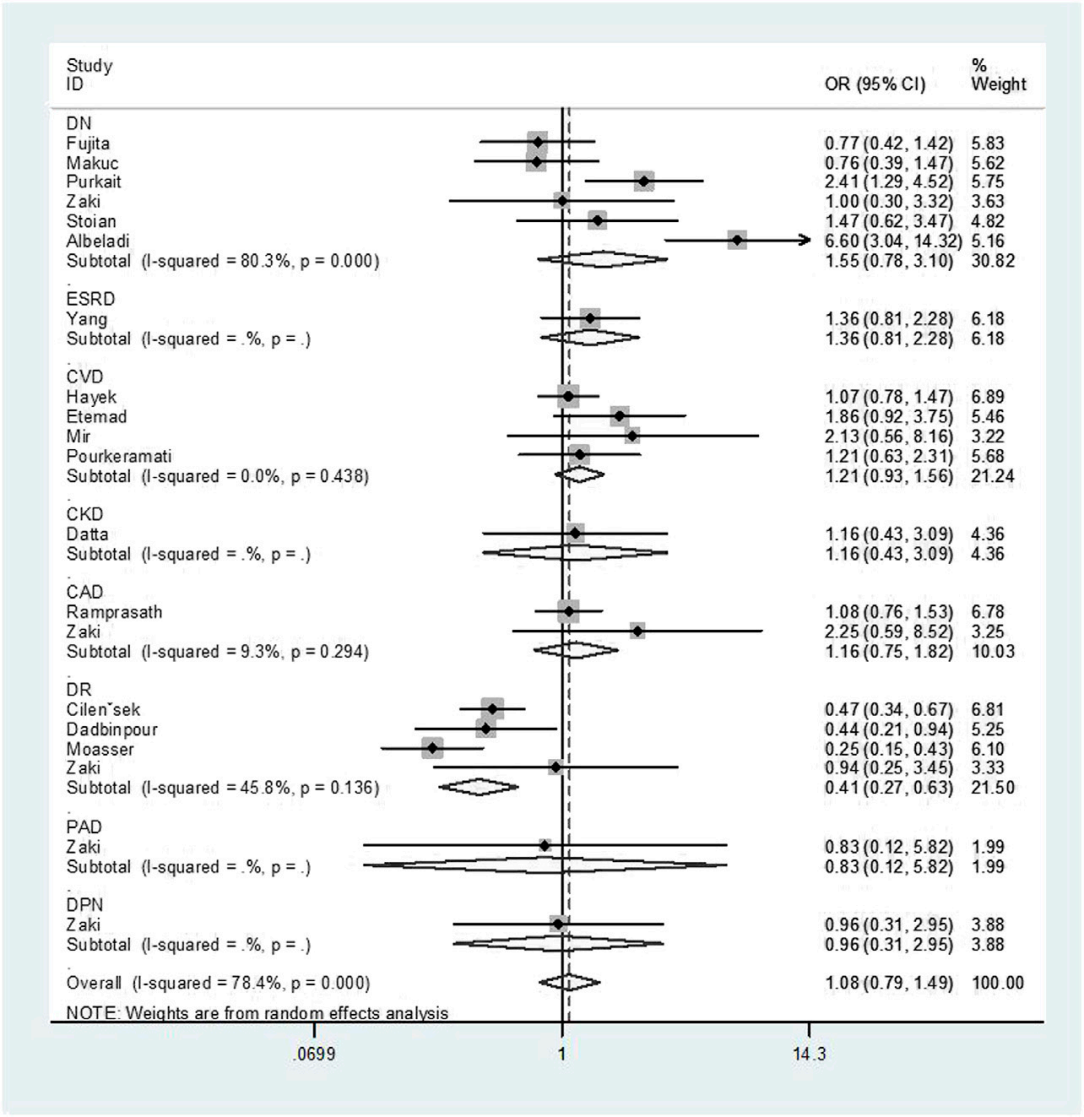
Forest plot of the association between GSTT1 polymorphism and T2DM complications risk.

**FIGURE 3:**
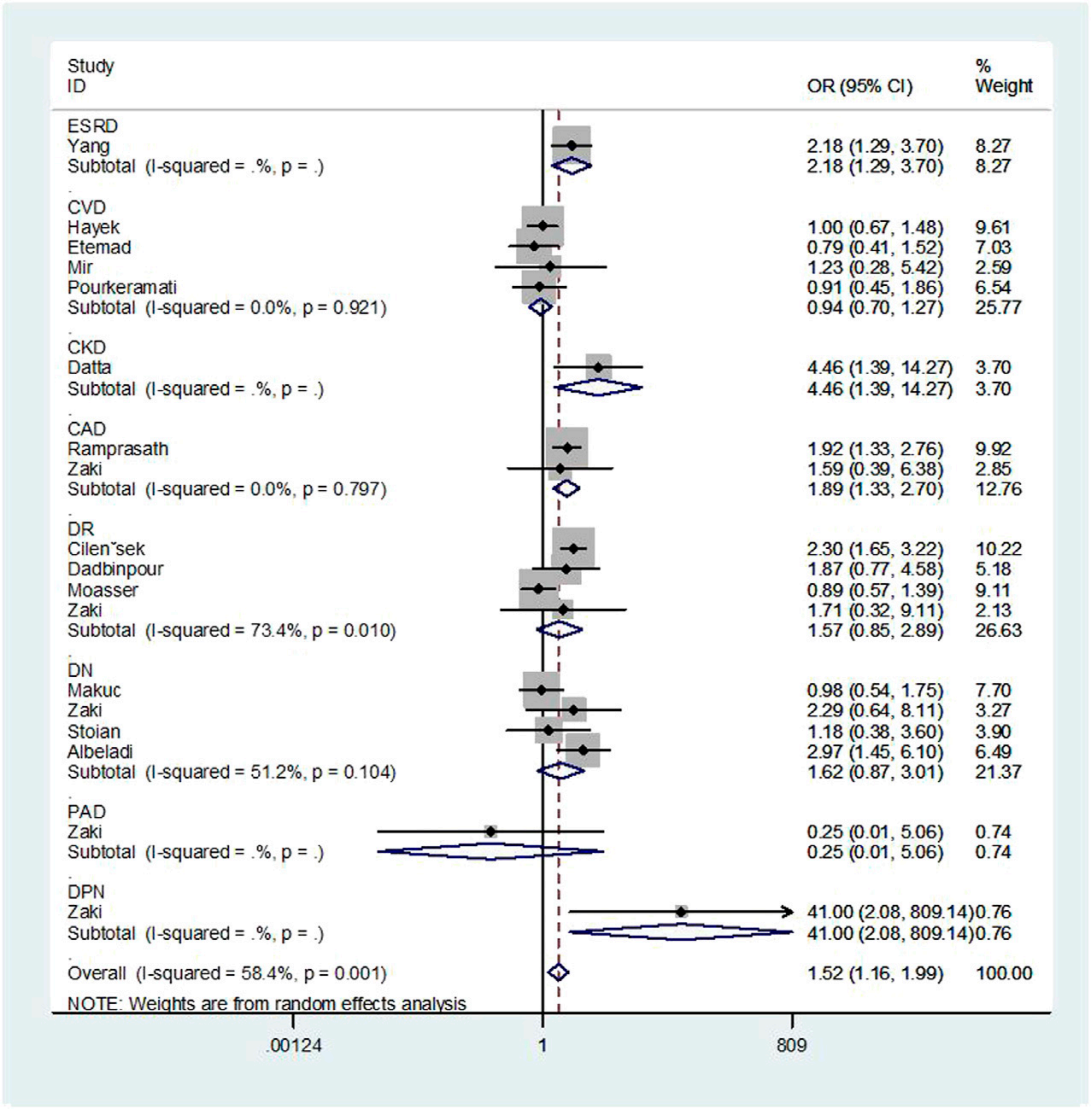
Forest plot of the association between GSTM1 polymorphism and T2DM complications risk.

**FIGURE 4:**
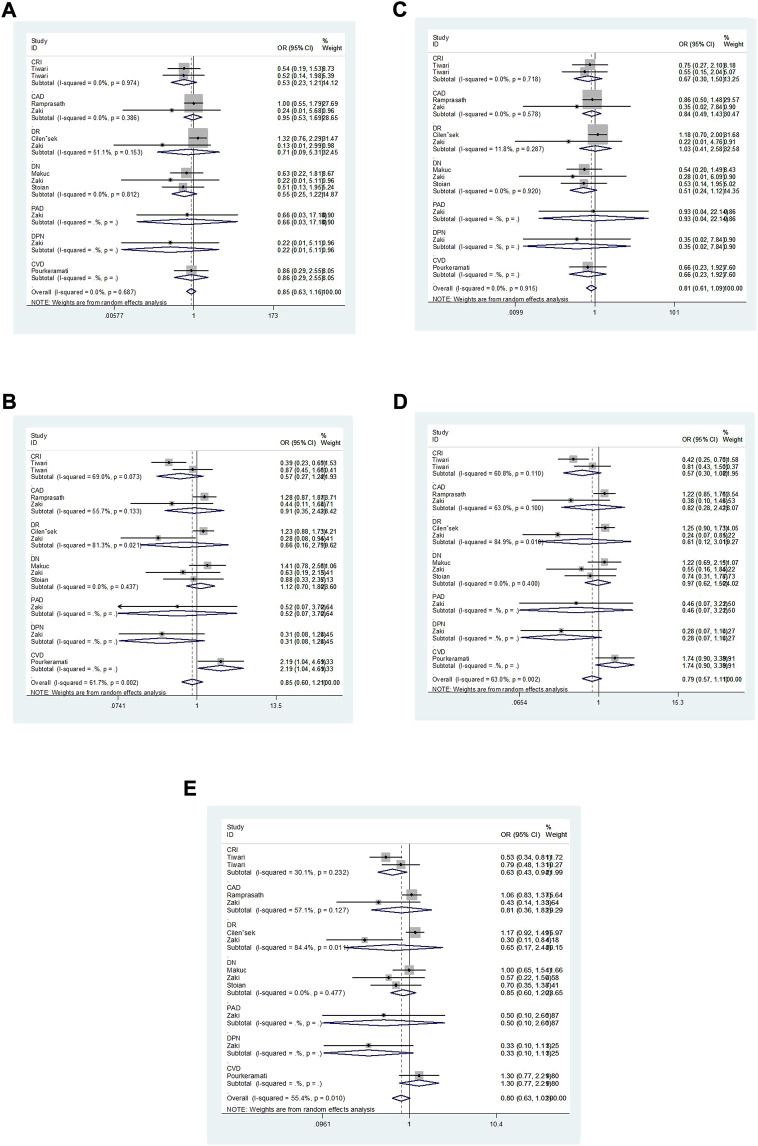
Forest plot of the association between GSTPI polymorphism and T2DM complications risk [**(A)**: VV vs. II; **(B)**: IV vs. II; **(C)**: VV vs. IV + II; **(D)**: VV + IV vs. II; **(E)**: V vs. I].

Overall, a notable association was discovered between the GSTT1 null genotype and an increased risk of T2DM complications (OR = 1.52, 95% CI: 1.16–1.99, Figure 2). No association was found between GSTM1 and GSTP1 polymorphisms and the risk of T2DM complications (Figures 3, 4).

### Heterogeneity and sensitivity analyses

We assessed heterogeneity source by applying a meta-regression analysis. The source of heterogeneity for the GSTT1 null genotype and the combined effects of GSTT1 and GSTP1 was revealed to be the source of controls (*p* = 0.028 and model 1: *p* = 0.037). HWE (model 7: *p* = 0.045) was the source of heterogeneity between the combined effects of GSTM1, GSTT1, and GSTP1 gene and T2DM risk.

Sensitivity analysis was estimated by applying three methods. First, results did not change when removing a single study each time to appraise the robustness. Second, there was no significant connection individual GSTM1 and GSTT1 polymorphisms when studies of low quality and non-matching were eliminated (Tables 1, 2). Moreover, concerning the GSTP1 polymorphism, a noteworthy association with T2DM risk was found in the overall analysis (Val vs. IIe: OR = 1.39, 95% CI = 1.01–1.93, Table 3), Indians (Val/Val vs. IIe/IIe: OR = 2.21, 95% CI = 1.51–3.24, IIe/Val vs. IIe/IIe: OR = 1.53, 95% CI = 1.09–2.15, Val/Val vs. IIe/IIe + IIe/Val: OR = 1.88, 95% CI = 1.30–2.73, Val/Val + IIe/Val vs. IIe/IIe: OR = 1.65, 95% CI = 1.21–2.26, Val vs. IIe: OR = 1.54, 95% CI = 1.18–2.00), population-based studies (Val/Val vs. IIe/IIe: OR = 2.56, 95% CI = 1.47–4.44, Val/Val vs. IIe/IIe + IIe/Val: OR = 2.29, 95% CI = 1.36–3.85, Val/Val + IIe/Val vs. IIe/IIe: OR = 1.51, 95% CI = 1.15–1.99, Val vs. IIe: OR = 1.48, 95% CI = 1.20–1.83) and Asia when we retained research of low quality and HWD in control.

When we restricted only high-quality studies, notably increased T2DM risk was found the combinations of GSTM1 and GSTT1 polymorphisms (model 3: OR = 3.40, 95% CI = 2.42–4.79; model 4: OR = 2.00, 95% CI = 1.54–2.59; model 5: OR = 1.39, 95% CI = 1.12–1.74; model 6: OR = 2.21, 95% CI = 1.40–3.50, Table 4). Significantly increased T2DM risk was observed the GSTM1-GSTT1 polymorphisms (model 2: OR = 2.34, 95% CI = 1.68–3.26; model 3: OR = 1.42, 95% CI = 1.04–1.94; model 4: OR = 1.56, 95% CI = 1.25–1.94; model 5: OR = 1.27, 95% CI = 1.11–1.46; model 6: OR = 1.69, 95% CI = 1.31–2.19, Table 4) When we excluded HWD.

When we removed low-quality studies, significantly increased T2DM risk was found in overall analysis (model 1: OR = 2.57, 95% CI = 1.53–4.31, Table 5), and when we removed HWD, significantly association was revealed in the overall analysis (model 1: OR = 1.72, 95% CI = 1.15–2.58; model 3: OR = 1.30, 95% CI = 1.01–1.67; model 4: OR = 2.24, 95% CI = 1.41–3.54; model 5: OR = 1.42, 95% CI = 1.01–2.01; model 6: OR = 1.48, 95% CI = 1.03–2.15) combined effects on GSTM1 and GSTP1 polymorphisms.

Concerning the combination effects of GSTM1, GSTT1, and GSTP1 IIe105Val polymorphisms, considerably increased T2DM risk was observed (model 1: OR = 2.81, 95% CI = 1.02–3.22, model 9: OR = 1.75, 95% CI = 1.00–3.07, model 10: OR = 2.24, 95% CI = 1.32–3.80, Table 7) when we excluded low-quality studies. Significantly increased T2DM risk was revealed (model 1: OR = 1.61, 95% CI = 1.15–2.25, model 9: OR = 1.87, 95% CI = 1.12–3.11, model 10: OR = 2.19, 95% CI = 1.35–3.54) when HWD was removed in overall analysis. On the contrary, no notable was detected on the combined effects of GSTT1 and GSTP1 polymorphisms (Table 6).

### Publication bias

No publication bias was observed between GSTs polymorphisms with T2DM risk through the Begg’s funnel plot and Egger’s test (Figures not shown).

## Discussion

Oxidative stress is considered to play an important role in the pathogenesis of diabetes mellitus and related complications. GST is a member of phase II metabolic isoenzyme group, which has the effect of regulating various cytotoxicity, genotoxicity and antioxidant. The risk genotypes expressed by each gene (GSTM1 null, GSTT1 null, and GSTP1 Val/Val) may decrease the antioxidant activity of the enzyme, thus increasing the susceptibility of T2DM and other diseases. There was much important evidence showing that GSTM1, GSTT1, and GSTP1 gene polymorphisms were potential genetic factors for T2DM. Yet, previous research remains contradictory. In addition, seven previously published meta-analyses also had certain limitations, therefore the association of the GSTs polymorphisms with T2DM risk needs to be further evaluated. Furthermore, to our knowledge, this research is the first large-scale meta-analysis to investigate the relationship between individual and combined GSTM1, GSTT1, and GSTP1 genes polymorphism of T2DM susceptibility, and utilizing FPRP and BFDP to explore the validity of positive results.

Overall, on the individual and combined effects of GSTM1, GSTT1, and GSTP1 polymorphisms, a statistically significantly increased T2DM risk was found. We found that GSTT1 null genotype increased the risk of T2DM complications. However, GSTM1 and GSTP1 polymorphism were not associated with T2DM complications risk. The GSTM1 null genotype was correlated to a significantly increased risk of T2DM in Caucasians, Asia, hospital-based studies, and non-diabetic controls. The GSTT1 null genotype was connected with an increased risk of T2DM in Asians, Indians, Asia, and so on. GSTP1 IIe105Val polymorphism was correlated with an increased risk of T2DM in Indians, Asia, population-based studies, and healthy controls. The GSTM1-GSTT1 double null genotype significantly increased the risk of T2DM in both subgroup and sensitivity analysis. The effects of GSTM1-GSTP1 gene polymorphisms were related to the risk of T2DM in Caucasians and matching. However, no relationship was identified between GSTT1-GSTP1 polymorphisms and T2DM susceptibility, and, interestingly, Indians had a significantly increased risk. For the triple genotype combinations, we also discovered an association with T2DM. These findings showed that the same gene has variable susceptibility to T2DM in different race groups. It is worth considering that the obvious inconsistencies could be due to sample size, GSTs prevalence and environmental factors (lifestyle). Additionally, because T2DM is a chronic metabolic disease with multiple factors and genes, and different genetic backgrounds may lead to such differences. We found that GSTs gene polymorphism increased the risk of T2DM in Asia, but not in Europe. Most of the original research were conducted in Asia, which may lead to this difference. Therefore, a larger sample size is needed to study the relationship between GSTs gene polymorphism and T2DM in Europe, America, Africa and other geographic regions. The GSTM1- GSTT1, GSTM1- GSTP1 and GSTM1-GSTT1-GSTP1 gene polymorphisms combination are all associated with the increased susceptibility of T2DM. These results indicate that polygenes may have cumulative effects, but the potential gene-gene interactions still need to be studied with a larger sample size.

However, the present study used several subgroups and diverse genetic models, resulting in multiple comparisons, which necessitated adjusting the pooled *p* value (Attia et al., 2003). FPRP is regarded an acceptable tool for calculating the likelihood of positive findings in multiple hypothesis testing of molecular epidemiology investigations (Wacholder et al., 2004). Wakefield provided a much more reliable Bayesian metric of false positives in genetic epidemiology research (Wakefield, 2008). Potential errors and bias may be caused by genotyping errors or high inter-study heterogeneity (I^2^ > 50%) (Page et al., 2003; Clayton et al., 2005; Pompanon et al., 2005; Balding, 2006; Zhang et al., 2013), while large volumes evidence (statistical power >80%) have stricter statistical significance levels or lower false-discovery rate (Benjamini and Hochberg, 1995). Therefore, we consider using FPRP tests, BFDP tests and the Venice criteria to evaluate the false findings in present studies. The current meta-analysis found that all substantial relationship were deemed as less credible during did the reliability analysis. According to the results of the meta-regression analysis, the HWD studies and sources of controls were the main causes of heterogeneity. Bias and errors are prevalent in some low-quality, small-sample HWD studies, making the conclusions of these original studies undependable, molecular biology and disease susceptibility study, in particular. Furthermore, research with a small sample size and substantial results may be likely to swallow than studies with negative outcomes. When they produce positive results, however, their research might not have been rigorous and typically of poor quality.

Therefore, we assessed the sensitivity analysis using matching, high-quality, and HWE.

These three GSTs gene polymorphisms and their interactions were discovered to be strongly related to diabetes susceptibility in this investigation. From 2012 to 2019, a total of five related meta-studies were published. Recent meta-studies by Nath et al. (2019) showed that the GSTM1, GSTT1 null genotype and GSTM1-GSTT1 double null genotype increased the risk of T2DM in both Asian and Caucasian populations, the results are consistent with this study. An analysis of eleven studies by Yi et al. (2013) revealed that the GSTM1 gene polymorphisms increases the susceptibility to T2DM in Asians, Caucasians, and Africans. However, Saadat (2017) selected 18 studies (2,595 patients and 2,888 controls) and suggested that GSTP1 IIe105Val polymorphism was not connected with the risk of T2DM, which was contrary to the results of this study. Tang et al. (2013) also analyzed the three genes and suggested that GSTM1 and GSTT1 null genotypes were correlated to an increased risk of T2DM in Asians, while GSTP1 gene polymorphism was not. For the GSTP1 gene, the opposite result was obtained. Three related meta-analysis were published from 2015 to 2019. Nath et al. showed that GSTT1 null genotype increased the risk of T2DM related complications, but GSTM1 null genotype did not, which was consistent with this study. On the contrary, Orlewski and Orlewska (2015) found that GSTM1 gene polymorphisms increased the risk of diabetic nephropathy, while GSTT1 and GSTP1 gene polymorphisms were not statistically significant. Sun et al. (2015) suggested that a strong association GSTM1 and GSTP1 polymorphisms and diabetic retinopathy susceptibility. Further investigate the effect of gene-gene interaction on T2DM, two previous studies reported the correlation between GSTM1-GSTT1 gene polymorphism and T2DM susceptibility, but did not study the combined effect between the remaining genes. In this study, all except GSTT1-GSTP1 combination were found to be related. Moreover, these published meta-analyses are obviously inconsistent with current meta-analyses in terms of racial classification. The variability in results may be due to a variety of factors, such as small sample sizes, ethnic differences, failure to assess quality in most eligible studies and failure to develop a more complete genetic model.

There are several advantages to the newer meta-analysis. First, The FPRP, BFDP test, and Venice criterion were used to assess the credibility with positive outcomes. Secondly, quality assessment of qualified research is carried out. Third, the sample size was much larger and more detailed data and information were collected. Fourth, quality assessment of qualified research. Fifthly, a relatively complete genetic model is established. Sixth, more subgroup analyses were performed. Seventh, to our knowledge, this is the first meta-analysis to explore the combined effects of GSTM1-GSTP1, GSTT1-GSTP1, and GSTM1-GSTT1-GSTP1 gene polymorphisms on the risk of T2DM. There are some limitations of the present meta-analysis. To begin with, latest meta-analyses only include published articles, and positive results are frequently more likely to be published than negative consequences. The zero effect of GSTM1 may be overestimated if negative results are included. Second, because we could not calculate the HWE of these two genes, we did not evaluate whether the genotype distributions of GSTM1 and GSTT1 polymorphisms in the control group was in HWE. Third, heterogeneity between studies was much large in overall and among several subgroups. According to the results of the meta-regression analysis, the HWD studies and sources of controls were the main causes of heterogeneity, and adequate statistical correction and analysis did not significantly improve the heterogeneity. The source of heterogeneity may also be due to some other factors, such as study area, lifestyle and so on. Fourth, we conducted a subgroup analysis of race and region, and population heterogeneity has been resolved. However, T2DM is closely associated with the environment, the original study did not provide data on lifestyle, so we failed to solve the heterogeneity caused by lifestyle, and did not explore the effect of the gene-environment combination. Fifth, we failed to further investigate the effect of GSTs gene polymorphisms on the risk of T2DM related complications.

In conclusion, the current study revealed the relationship between GSTM1, GSTT1, and GSTP1 gene polymorphisms with the risk of T2DM, which may be attributed to false positive results rather than actual correlations or biological variables. Larger epidemiological studies should be carried out in the future to confirm or deny our findings.

## Data Availability

The original contributions presented in the study are included in the article/Supplementary Material, further inquiries can be directed to the corresponding authors.
