# Correction: Individual and combined effects of the GSTM1, GSTT1, and GSTP1 polymorphisms on type 2 diabetes mellitus risk: a systematic review and meta-analysis

**DOI:** 10.3389/fgene.2025.1749934

**Published:** 2025-12-01

**Authors:** Liang-shu Liu, Di Wang, Ru Tang, Qi Wang, Lu Zheng, Jian Wei, Yan Li, Xiao-feng He

**Affiliations:** 1 Changzhi Medical College, Changzhi, Shanxi, China; 2 Department of Endocrinology, Heping Hospital Affiliated to Changzhi Medical College, Changzhi, Shanxi, China; 3 Department of Epidemiology, School of Public Health to Southern Medical University, Guangzhou, Guangdong, China; 4 Institute of Evidence-Based Medicine, Heping Hospital Affiliated to Changzhi Medical College, Changzhi, China

**Keywords:** meta-analysis, genetic polymorphism, GSTM1, GSTP1, GSTT1, T2DM

An incorrect grant number was provided for Shanxi Basic Research Program in the **Funding** statement. The correct numbers are: 20210302123076; 20210302123012.

The original article has been updated.

