# Supplementary material for: Individual and combined effects of the GSTM1, GSTT1, and GSTP1 polymorphisms on type 2 diabetes mellitus risk: A systematic review and meta-analysis
Source: Front Genet. 2022 Nov 7;13:959291. doi: 10.3389/fgene.2022.959291 (PMC9676647; doi:10.3389/fgene.2022.959291)
Supplement: Supplementary file 1 [file Table4.doc]

**Supplemental Table 4 Genotype frequencies of the combined effects of *GSTM1* present/null and *GSTT1* present/null between T2DM and control groups**

| First author/Year | Country | Ethnicity | SC | Genotype | | | | | | | | | | | |
| --- | --- | --- | --- | --- | --- | --- | --- | --- | --- | --- | --- | --- | --- | --- | --- |
|  |  |  |  | A | | B | | C | | D | | E | | F | |
|  |  |  |  | Cases | Controls | Cases | Controls | Cases | Controls | Cases | Controls | Cases | Controls | Cases | Controls |
| Hori 2007 | Japan | Asian | HB | 16 | 92 | 21 | 97 | 19 | 119 | 7 | 98 | 35 | 211 | 42 | 309 |
| Yalin 2007 | Turkey | Caucasian | HB | 8 | 16 | 13 | 6 | 50 | 27 | 27 | 49 | 58 | 43 | 85 | 92 |
| Bid 2010 | India | Indian | HB | NA | NA | 5 | 8 | NA | NA | 40 | 110 | 55 | 82 | 95 | 192 |
| Datta 2010 | India | Indian | PB | 18 | 7 | 25 | 3 | 23 | 11 | 34 | 29 | 41 | 18 | 75 | 47 |
| Amer 2011 | Egypt | Caucasian | PB | NA | NA | 19 | 12 | NA | NA | 22 | 44 | 59 | 44 | 81 | 88 |
| Gönül 2012 | Turkey | Caucasian | HB | NA | NA | 15 | 4 | NA | NA | 15 | 24 | NA | NA | NA | NA |
| Moasser 2012 | Iran | Caucasian | HB | 9 | 15 | 34 | 20 | 76 | 66 | 52 | 68 | 85 | 81 | 137 | 149 |
| Mastana 2013 | India | Indian | PB | NA | NA | 63 | 24 | NA | NA | 126 | 184 | 132 | 101 | 258 | 285 |
| Pinheiro 2013 | Brazil | Mixed | HB | 27 | 12 | 8 | 6 | 42 | 58 | 43 | 71 | 69 | 70 | 112 | 141 |
| Vats 2013 | India | Indian | HB | 32 | 33 | 25 | 15 | 32 | 44 | 110 | 109 | 64 | 77 | 174 | 186 |
| Moasser 2014 | Iran | Caucasian | HB | 38 | 17 | 67 | 23 | 166 | 75 | 133 | 86 | 204 | 92 | 337 | 178 |
| Rao 2014 | India | Indian | HB | NA | NA | 50 | 33 | NA | NA | 116 | 115 | 78 | 80 | 194 | 195 |
| Zaki 2015 | Egypt | Caucasian | HB | NA | NA | 21 | 26 | NA | NA | 22 | 16 | 11 | 9 | 33 | 25 |
| Stoian 2015 | Romania | Caucasian | HB | 4 | 12 | 11 | 13 | 33 | 38 | 36 | 35 | 37 | 50 | 73 | 85 |
| Etemad 2016 | Malaysia | Asian | HB | 22 | 22 | 42 | 24 | 57 | 65 | 30 | 25 | 79 | 87 | 109 | 112 |
| Azarova 2018 | Russia | Caucasian | HB | NA | NA | 57 | 22 | NA | NA | 68 | 94 | NA | NA | NA | NA |
| Jamil 2022 | Pakistan | Asian | HB | NA | NA | 30 | 7 | NA | NA | 80 | 86 | 134 | 102 | 214 | 188 |

NA = not available, ^a^ = IIe/Val + Val/Val, A = M1 *GSTM1* present/*GSTT1* null, B = *GSTM1* null/*GSTT1* null, C = *GSTM1* null/*GSTT1* present, D = *GSTM1* present/*GSTT1* present, E = *GSTM1* present/*GSTT1* null + *GSTM1* null/*GSTT1* present, F = *GSTM1* present/*GSTT1* present + *GSTM1* present/*GSTT1* null + *GSTM1* null/*GSTT1* present
