# Supplementary material for: Individual and combined effects of the GSTM1, GSTT1, and GSTP1 polymorphisms on type 2 diabetes mellitus risk: A systematic review and meta-analysis
Source: Front Genet. 2022 Nov 7;13:959291. doi: 10.3389/fgene.2022.959291 (PMC9676647; doi:10.3389/fgene.2022.959291)
Supplement: Supplementary file 2 [file Table6.doc]

**Supplemental Table 6 Genotype frequencies of the combined effects of *GSTT1* present/null and *GSTP1* IIe105Val between T2DM and control groups**

| First author/Year | Ethnicity | *GSTT1* present/ *GSTP1* IIe/IIe | | *GSTT1* null/ *GSTP1* IIe/IIe | | *GSTT1* present/ *GSTP1* Val ^1^ | | Total one risk genotype | | *GSTT1* null/*GSTP1* Val ^1^ | | All risk genotypes | |
| --- | --- | --- | --- | --- | --- | --- | --- | --- | --- | --- | --- | --- | --- |
|  |  | Case | Control | Case | Control | Case | Control | Case | Control | Case | Control | Case | Control |
| Yalin 2007 | Caucasian | 36 | 37 | 13 | 7 | 41 | 39 | 54 | 46 | 8 | 15 | 62 | 61 |
| Bid 2010 | Indian | 72 | 104 | 9 | 15 | 16 | 68 | 25 | 83 | 3 | 13 | 28 | 96 |
| Mastana 2013 | Indian | 101 | 116 | 32 | 6 | 121 | 127 | 153 | 133 | 81 | 31 | 234 | 164 |
| Vats 2013 | Indian | 112 | 125 | 31 | 43 | 35 | 28 | 66 | 71 | 24 | 5 | 90 | 76 |
| Rao 2014 | Indian | 58 | 62 | 11 | 10 | 12 | 16 | 23 | 26 | 43 | 23 | 66 | 49 |
| Zaki 2015 | Caucasian | 23 | 24 | 6 | 14 | 13 | 8 | 19 | 22 | 12 | 5 | 31 | 27 |
| Stoian 2015 | Caucasian | 40 | 52 | 9 | 20 | 29 | 21 | 38 | 41 | 6 | 5 | 44 | 46 |
| Azarova 2018 | Caucasian | NA | NA | NA | NA | NA | NA | NA | NA | 19 | 4 | NA | NA |
| Jamil 2022 | Asian | 156 | 150 | 47 | 28 | NA | NA | NA | NA | NA | NA | NA | NA |

NA = not available, Val^1^ = IIe/Val + Val/Val, Total one risk genotype = *GSTT1* null/ *GSTP1* IIe/IIe + *GSTT1* present/ *GSTP1* Val ^1^ , All risk genotypes = *GSTT1*

null/ *GSTP1* IIe/IIe + *GSTT1* present/ *GSTP1* Val ^1^ + *GSTT1* null/*GSTP1* Val ^1^
