# Supplementary material for: Individual and combined effects of the GSTM1, GSTT1, and GSTP1 polymorphisms on type 2 diabetes mellitus risk: A systematic review and meta-analysis
Source: Front Genet. 2022 Nov 7;13:959291. doi: 10.3389/fgene.2022.959291 (PMC9676647; doi:10.3389/fgene.2022.959291)
Supplement: Supplementary file 5 [file Table7.doc]

**Supplemental Table 7 Genotype frequencies of the combined effects of *GSTM1*, *GSTT1* and *GSTP1* IIe105Val polymorphisms between T2DM and control groups**

| First author/Year | A | | B | | C | | D | | E | | F | | G | | H | | I | | J | |
| --- | --- | --- | --- | --- | --- | --- | --- | --- | --- | --- | --- | --- | --- | --- | --- | --- | --- | --- | --- | --- |
|  | Case | Control | Case | Control | Case | Control | Case | Control | Case | Control | Case | Control | Case | Control | Case | Control | Case | Control | Case | Control |
| Yalin 2007 | 14 | 20 | 22 | 17 | 5 | 5 | 13 | 29 | 40 | 51 | 8 | 2 | 28 | 10 | 3 | 11 | 39 | 23 | 5 | 4 |
| Bid 2010 | 33 | 72 | 39 | 35 | 5 | 8 | 6 | 41 | 50 | 84 | 4 | 6 | 10 | 27 | 2 | 9 | 16 | 42 | 1 | 2 |
| Mastana 2013 | 161 | 98 | 40 | 18 | 11 | 19 | 65 | 85 | 116 | 122 | 7 | 5 | 56 | 42 | 25 | 22 | 88 | 69 | 56 | 19 |
| Vats 2013 | 84 | 92 | 28 | 33 | 18 | 29 | 26 | 17 | 72 | 79 | 13 | 14 | 9 | 11 | 13 | 4 | 35 | 29 | 11 | 1 |
| Rao 2014 | 79 | 93 | 19 | 14 | 32 | 37 | 16 | 28 | 67 | 79 | 27 | 24 | 15 | 16 | 27 | 8 | 69 | 48 | 29 | 8 |
| Stoian 2015 | 21 | 24 | 19 | 28 | 3 | 8 | 15 | 11 | 37 | 47 | 6 | 12 | 14 | 10 | 1 | 4 | 21 | 26 | 5 | 1 |
| Jamil 2022 | 67 | 81 | 86 | 58 | 21 | 24 | NA | NA | NA | NA | 26 | 3 | NA | NA | NA | NA | NA | NA | NA | NA |

NA = not available, A = *M1* Present/*T1* Present/*P1* IIe/IIe, B = *M1* Null/*T1* Present/*P1* IIe/IIe, C = *M1* Present/*T1* Null/*P1* IIe/IIe, D = *M1* Present/*T1* Present/*P1* Val ^1^, E = *M1* Null/*T1* Present/*P1* IIe/IIe + *M1* Present/*T1* Null/*P1* IIe/IIe + *M1* Present/*T1* Present/*P1* Val ^1^, F = *M1* Null/*T1* Null/*P1* IIe/IIe, G = *M1* Null/*T1* Present/*P1* Val ^1^, H = *M1* Present/*T1* Null/*P1* Val ^1^, I = *M1* Null/*T1* Null/*P1* IIe/IIe + *M1* Null/*T1* Present/*P1* Val ^1^ + *M1* Present/*T1* Null/*P1* Val ^1^, J = *M1* Null/*T1* Null/*P1* Val^1^, Val^1^: IIe/Val + Val/Val
