# Supplementary material for: Individual and combined effects of the GSTM1, GSTT1, and GSTP1 polymorphisms on type 2 diabetes mellitus risk: A systematic review and meta-analysis
Source: Front Genet. 2022 Nov 7;13:959291. doi: 10.3389/fgene.2022.959291 (PMC9676647; doi:10.3389/fgene.2022.959291)
Supplement: Supplementary file 6 [file Table5.doc]

**Supplemental Table 5 Genotype frequencies of the combined effects of *GSTM1* present/null and *GSTP1* IIe105Val polymorphism between T2DM and control groups**

| First author/Year | Ethnicity | *GSTM1* present/ *GSTP1* IIe/IIe | | *GSTM1* null/ *GSTP1* IIe/IIe | | *GSTM1* present/*GSTP1* Val ^1^ | | Total one risk genotype | | *GSTM1* null/ *GSTP1* Val ^1^ | | All risk genotypes | |
| --- | --- | --- | --- | --- | --- | --- | --- | --- | --- | --- | --- | --- | --- |
|  |  | Case | Control | Case | Control | Case | Control | Case | Control | Case | Control | Case | Control |
| Yalin 2007 | Caucasian | 19 | 25 | 30 | 19 | 16 | 40 | 46 | 59 | 33 | 14 | 79 | 73 |
| Bid 2010 | Indian | 39 | 77 | 42 | 42 | 8 | 50 | 50 | 92 | 11 | 31 | 61 | 123 |
| Mastana 2013 | Indian | 72 | 118 | 47 | 23 | 90 | 106 | 137 | 129 | 112 | 61 | 249 | 190 |
| Vats 2013 | Indian | 102 | 121 | 41 | 47 | 40 | 21 | 81 | 68 | 21 | 12 | 102 | 80 |
| Rao 2014 | Indian | 50 | 35 | 30 | 33 | 25 | 29 | 55 | 62 | 15 | 20 | 70 | 82 |
| Zaki 2015 | Caucasian | 14 | 16 | 15 | 22 | 14 | 9 | 29 | 31 | 11 | 4 | 40 | 35 |
| Stoian 2015 | Caucasian | 24 | 32 | 25 | 40 | 16 | 15 | 41 | 55 | 19 | 11 | 60 | 66 |
| Azarova 2018 | Caucasian | 44 | 60 | NA | NA | NA | NA | NA | NA | 45 | 23 | NA | NA |
| Jamil 2022 | Asian | 88 | 107 | 110 | 70 | NA | NA | NA | NA | NA | NA | NA | NA |

Val ^1^ = IIe/Val + Val/Val, Total one risk genotype = *GSTM1* null/ *GSTP1* IIe/IIe + *GSTM1* present/*GSTP1* Val ^1^, All risk genotypes = *GSTM1* null/ *GSTP1* IIe/IIe + *GSTM1* present/*GSTP1* Val ^1^ + *GSTM1* null/ *GSTP1* Val ^1^, NA = not available
