# Supplementary material for: Individual and combined effects of the GSTM1, GSTT1, and GSTP1 polymorphisms on type 2 diabetes mellitus risk: A systematic review and meta-analysis
Source: Front Genet. 2022 Nov 7;13:959291. doi: 10.3389/fgene.2022.959291 (PMC9676647; doi:10.3389/fgene.2022.959291)
Supplement: Supplementary file 7 [file Table2.docx]

**Supplemental Table 2** Scale for quality assessment of molecular association studies of T2DM

| Criterion | Score |
| --- | --- |
| Source of case | |
| Selected from population | 2 |
| Selected from hospital | 1 |
| Not described | 0 |
| Source of control | |
| Population-based | 2 |
| Hospital-based | 1 |
| Not described | 0 |
| Ascertainment of cancer | |
| Diagnosis of T2DM by WHO or ADA | 2 |
| Diagnosis of T2DM by patient medical record | 1 |
| Not described | 0 |
| Ascertainment of control | |
| Controls were tested to screen out T2DM | 2 |
| Controls were subjects who did not report T2DM, no objective testing | 1 |
| Not described | 0 |
| Matching | |
| Controls matched with cases by age and sex | 2 |
| Controls matched with cases only by age or sex | 1 |
| Not matched or not described | 0 |
| Genotyping examination | |
| Genotyping done blindly and quality control | 2 |
| Only genotyping done blindly or quality control | 1 |
| Unblinded and without quality control | 0 |
| HWE | |
| HWE in the control group | 2 |
| HWD in the control group | 0 |
| Association assessment | |
| Assess association between genotypes andT2DM with appropriate statistics and adjustment for confounders | 2 |
| Assess association between genotypes andT2DM with appropriate statistics without adjustment for confounders | 1 |
| Inappropriate statistics used | 0 |
| Total sample size |  |
| ≥ 200 | 2 |
| <200 | 0 |

HWE: Hardy-Weinberg equilibrium, HWD: Hardy-Weinberg disequilibrium, T2DM: type 2 diabetes mellitus
